# Galectin-3 and sST2: associations to the echocardiographic markers of the myocardial mechanics in systemic sclerosis – a pilot study

**DOI:** 10.1186/s12947-022-00272-7

**Published:** 2022-01-18

**Authors:** Vivien Vértes, Adél Porpáczy, Ágnes Nógrádi, Margit Tőkés-Füzesi, Máté Hajdu, László Czirják, András Komócsi, Réka Faludi

**Affiliations:** 1 Heart Institute, Medical School, University of Pécs, Ifjúság út 13, H-7624 Pécs, Hungary; 2 Department of Laboratory Medicine, Medical School, University of Pécs, Ifjúság út 13, H-7624 Pécs, Hungary; 3 Department of Rheumatology and Immunology, Medical School, University of Pécs, Akác u. 1, H-7632 Pécs, Hungary

**Keywords:** Systemic sclerosis, Myocardial fibrosis, Galectin-3, sST2, Global longitudinal strain, Diastolic dysfunction

## Abstract

**Background:**

Progressive cardiac fibrosis is the central aspect of the myocardial involvement in systemic sclerosis (SSc). We hypothesized that circulating biomarkers of the cardiac fibrosis may be useful in the early diagnosis of the cardiac manifestation in this disease. Thus, we investigated the potential correlations between the levels of galectin-3, soluble suppression of tumorigenicity-2 (sST2) and the echocardiographic markers of the myocardial mechanics in SSc patients.

**Methods:**

Forty patients (57.3 ± 13.7 years, 36 female) were investigated. In addition to the conventional echocardiography, tissue Doppler and speckle tracking-derived strain techniques were used to assess the function of both ventricles and atria. To estimate the correlations between galectin-3 and sST2 levels and the echocardiographic variables, partial correlation method was used with age as correcting factor.

**Results:**

In age adjusted analysis galectin-3 level showed significant correlation with left ventricular global longitudinal strain (r = 0.460, *p* = 0.005); grade of left ventricular diastolic dysfunction (r = 0.394, *p* = 0.013); septal e’ (r = − 0.369, *p* = 0.021); septal E/e’ (r = 0.380, *p* = 0.017) and with the grade of mitral regurgitation (r = 0.323, *p* = 0.048). No significant correlation was found between sST2 levels and the echocardiographic variables.

**Conclusions:**

Galectin-3 levels, but not sST2 levels show significant correlation with the parameters of the left ventricular systolic and diastolic function. Galectin-3 may be a useful biomarker for the screening and early diagnosis of SSc patients with cardiac involvement.

## Introduction

Systemic sclerosis (SSc) is a connective tissue disease characterized by vascular abnormalities and diffuse fibrosis of the skin and various internal organs [1]. Although its pathogenesis is still not fully elucidated, recurrent ischaemic episodes resulting from coronary microcirculatory abnormalities may be responsible for the myocardial fibrosis [2], which represents the primary myocardial involvement of the disease [3]. Myocardial fibrosis may lead to left ventricular (LV) diastolic dysfunction, which is highly prevalent in SSc [4–6]. Although impaired LV ejection fraction is not common in this disease [7], myocardial fibrosis may eventuate in subclinically impaired LV systolic function, as it was proved by speckle tracking-derived global longitudinal strain (GLS) data [8, 9]. Subclinical right ventricular (RV) dysfunction was also proved, even in SSc patients without manifest pulmonary hypertension, using tissue Doppler or speckle tracking measurements [10–12]. Besides, impaired left (LA) and right atrial (RA) function have recently been reported in SSc, by the help of speckle tracking technique [10, 13–15].

Cardiac involvement implies poor prognosis in SSc [16, 17], thus its early, biomarker-based screening would be crucial. Utility of the well know cardiac biomarker, N-terminal pro–B-type natriuretic peptide (NT-proBNP) has already been proved in SSc [3, 18, 19]. In contrast, the potential clinical value of the circulating biomarkers reflecting myocardial fibrosis is less known in this disease.

Galectin-3 is a beta-galactoside-binding protein member of the lectin family that activates a variety of profibrotic factors, supports fibroblast proliferation and transformation, and mediates collagen production [20]. It plays an important role in cardiac remodelling by enhancing myocardial fibrosis [21]. In addition, it has been shown to be an independent predictor of outcome in heart failure [22, 23].

Soluble suppression of tumorigenicity-2 (sST2) is member of the interleukin-1 receptor family. It enhances myocardial hypertrophy and fibrosis by blocking the favourable influence of IL-33 [24]. Its prognostic value has been reported both in acute and chronic heart failure [24, 25].

Both galectin-3 and sST2 concentrations showed significant correlation with the echocardiographic markers of LV or LA size and function in heart failure patients [26–31]. Besides, in patients with pulmonary arterial hypertension, significant associations were reported between galectin-3 levels and the RV size and function whereas sST2 levels reflected disease severity [32–34].

Thus, in this study we aimed to investigate the potential associations between the levels of galectin-3 and sST2 and the echocardiographic markers of the myocardial mechanics in SSc patients.

## Patients and methods

### Study population

Our prospective study included 40 consecutive SSc patients diagnosed in the tertiary centre of the Department of Rheumatology and Immunology, University of Pécs. All enrolled cases fulfilled the updated ACR/EULAR classification criteria [35]. Patients with pulmonary arterial hypertension, atrial fibrillation, significant left sided valvular disease or known coronary artery disease were excluded from the study. Baseline clinical, laboratory and spirometry data were collected at enrolment. Duration of the disease was defined as time between the onset of the first non-Raynaud symptom of SSc and the inclusion, in years.

The study complied with the Declaration of Helsinki. The institutional ethics committee approved the study. Written informed consent was obtained from all patients.

### Echocardiography

All patients underwent echocardiographic examination performed by a single investigator using Philips EPIQ 7 ultrasound system (Philips Healthcare, Best, The Netherlands). M-mode and 2D data were collected: LV ejection fraction measured by biplane Simpson’s method; basal, mid-cavity, and longitudinal dimensions of the RV corrected for body surface area; tricuspid annular plane systolic excursion (TAPSE); RV fractional area change (RVFAC); maximal and minimal diameters of the inferior vena cava (IVC); collapsibility index of IVC (the percent decrease in the diameter during inspiration). LV mass was calculated according to the Devereux formula and corrected for body surface area (LV mass index). RV wall thickness was measured at end-diastole [36, 37]. Mitral and tricuspid regurgitations were evaluated according to the recent recommendation [38]. In addition to the spectral Doppler parameters of the trans-mitral and trans-tricuspid flow (E, A), myocardial systolic (S), early- (e’) and late- (a’) diastolic velocities were measured from apical four-chamber view at the lateral and septal border of the mitral annulus as well as on the tricuspid annulus using pulsed tissue Doppler imaging. Lateral and septal mitral myocardial velocities were averaged. Mitral and tricuspid E/A and E/e’ ratios were calculated [39]. Systolic pulmonary artery pressure was calculated from tricuspid regurgitation velocity added to the RA pressure. RA pressure (5 to 15 mmHg) was estimated using the diameter and collapsibility index of IVC [37]. Doppler measurements were obtained from ≥3 consecutive beats during end-expiratory apnoea. Elevated RV filling pressure was diagnosed if tricuspid E/e′ > 6 [36]. LV diastolic function was graded according to the current recommendation [39]. In “indeterminate” cases further parameters were also considered for the classification according to the 2020 revision of the recommendation [40].

### Strain measurements

For strain analysis, three consecutive heart cycles were recorded digitally. Care was taken to obtain true apical images using standard anatomic landmarks in each view. Foreshortening was avoided. Recordings were processed off-line, using a dedicated software (QLab, Philips Healthcare, Andover, MA, USA). Analysis was performed by a single investigator, blinded to the clinical and conventional echocardiographic data.

To estimate LV GLS, apical four-, three- and two-chamber movies were obtained using 2D echocardiography. The frame rate was set between 50 and 55 frames per second. Regional peak systolic longitudinal strain was determined in all 17 segments. LV GLS was automatically provided by the software as the average of the regional peak systolic longitudinal strain values.

For atrial speckle tracking analysis, atrium-focused apical four- and two-chamber view movies (four-chamber view for RA analysis) were obtained. The frame rate was set between 80 and 90 frames per second. The onset of R-wave on the electrocardiogram was used as zero-reference point of the strain analysis. Reservoir strain was defined as the peak systolic strain, just before mitral valve opening. This was followed by a plateau and a second late peak at the onset of the P-wave indicating the contractile strain. Conduit strain was calculated as the difference between reservoir and contractile strain. Results obtained in apical four- and two-chamber views were averaged for LA strain analysis [41].

Atrial volume curves were generated by the same software using the endocardial borders created for speckle tracking analysis. The following atrial volumes were obtained: maximal volume (Vmax) at the end of T wave on electrocardiogram, just before the opening of the mitral valve; minimal volume (Vmin) at QRS complex, just at the closure of the mitral valve; and preceding atrial contraction, at the beginning of P wave (Vp). LA volumes obtained in apical four- and two-chamber views were averaged. All volume values were corrected for body surface area (Vmax-, Vmin- and Vp index).

### Biomarkers

Blood samples were obtained immediately prior to the echocardiographic studies. Samples were stored at − 80 ∘C until testing.

Analysis of galectin-3 levels was performed using Human Galectin-3 Platinum ELISA kit developed by eBioscience (San Diego, CA, USA).

Analysis of sST2 levels was performed using Presage ST2 assay kit developed by Critical Diagnostics (San Diego, CA, USA).

Plasma concentrations of NT-proBNP were analysed by electrochemiluminescence immunoassay (Elecsys 2010 system, Roche Diagnostics, Mannheim, Germany).

### Statistical analysis

Categorical data were expressed as frequencies and percentages; continuous data were expressed as mean ± SD. Mann–Whitney U-test was used for comparisons of variables between two subgroups. Since concentrations of galectin-3, sST2 and NT-proBNP did not show normal distribution, logarithmic transformation was performed. Pearson bivariate method was used to investigate the correlations between ln galectin-3, ln sST2 and the other single variables. As age-related changes in the echocardiographic parameters are well known, in a second step, partial correlation method was applied using age as correcting factor. Partial regression plots were used to visualize these correlations. As creatinine or estimated glomerular filtration rate did not show correlation with galecin-3 or sST2 levels in our study, partial correlations were not adjusted for renal function. A *p* value of < 0.05 was considered significant. Data were analysed using IBM SPSS 25 statistical software.

## Results

A total of 40 SSc patients were enrolled into the study. Detailed clinical data of the patients are reported in Tables 1 and 2.

**Table 1:** Baseline characteristics of the systemic sclerosis population (*n* = 40) and their correlations with galectin-3 and sST2 levels

Clinical variable		Value	Correlation with ln galectin-3		Correlations with ln galectin-3, corrected for age		Correlations with ln sST2		Correlations with ln sST2, corrected for age	
			r	p	r	p	r	p	r	p
Age (years)		57.3 ± 13.7	0.059	0.719			−0.010	0.951		
Body surface area (m^2^)		1.8 ± 0.2	0.206	0.202	0.206	0.208	0.063	0.700	0.063	0.704
Disease duration (years)		8.0 ± 6.2	**0.406**	**0.009**	**0.406**	**0.010**	0.038	0.817	0.042	0.799
Modified Rodnan skin score		12.6 ± 9.6	−0.291	0.069	−0.286	0.077	0.200	0.216	0.205	0.210
New York Heart Association functional class	I n (%)	14 (35%)	0.179	0.269	0.171	0.297	−0.021	0.899	−0.018	0.912
	II n (%)	16 (40%)								
	III n (%)	10 (25%)								
6-min walking distance (m)		397.6 ± 78.3	−0.107	0.516	−.086	0.607	−0.001	0.996	0.001	0.996
Modified Borg dyspnoea index		1.8 ± 1.8	0.229	0.161	0.221	0.183	−0.234	0.151	−0.263	0.111
Forced expiratory volume in 1 s (%)		92.1 ± 15.5	**−0.320**	**0.044**	**−0.334**	**0.032**	−0.067	0.682	−0.066	0.689
Forced vital capacity (%)		94.9 ± 16.0	−0.246	0.126	−0.272	0.094	−0.157	0.333	−0.160	0.330
Diffusing capacity of carbon monoxide (%)		62.7 ± 16.2	**−0.318**	**0.045**	**−0.324**	**0.044**	0.106	0.515	0.123	0.456
Erythrocyte sedimentation rate (mm/h)		19.6 ± 13.5	−0.070	0.666	−0.079	0.633	0.099	0.545	0.101	0.541
C-reactive protein (mg/l)		4.2 ± 6.8	0.092	0.572	0.104	0.527	−0.118	0.468	−0.122	0.460
Creatinine (μmol/l)		63.8 ± 13.2	−0.013	0.936	−0.042	0.802	0.000	0.999	0.005	0.976
NT-proBNP (pg/ml; ln in correlations)		177.5 ± 148.6	0.094	0.566	0.081	0.623	−0.129	0.426	−0.131	0.425
Galectin-3 (ng/ml; ln in correlations)		12.9 ± 4.0					−0.217	0.178	−0.217	0.184
sST2 (ng/ml; ln in correlations)		28.5 ± 11.3	−0.217	0.178	−0.217	0.184				

Statistically significant *p*-values (*p* < 0.05) are formatted in bold

**Table 2:** Galectin-3 and sST2 levels in various subgroups of the study population

Clinical variable	Galectin-3		sST2	
	Level (ng/ml)	p*	Level (ng/ml)	p*
Female patients (*n* = 36; 90%)	13.2 ± 4.1	0.123	27.3 ± 9.8	0.207
Male patients (n = 4; 10%)	9.6 ± 0.2		40.1 ± 18.7	
Limited cutaneous form (*n* = 12; 30%)	12.9 ± 3.1	0.805	30.1 ± 9.5	0.389
Diffuse cutaneous form (*n* = 28; 70%)	12.9 ± 4.4		27.9 ± 12.1	
Anti-centromere antibody positive (*n* = 8; 20%)	11.3 ± 2.8	0.354	28.6 ± 9.4	0.871
Anti-centromere antibody negative (*n* = 32; 80%)	13.0 ± 4.1		29.1 ± 11.8	
Anti-topoisomerase antibody positive (*n* = 19; 47.5%)	12.8 ± 4.5	0.901	27.0 ± 8.6	0.989
Anti-topoisomerase antibody negative (*n* = 21; 52.5%)	12.8 ± 3.7		29.5 ± 13.6	
Patients with diabetes (n = 2; 5%)	13.4 ± 6.3	0.877	36.5 ± 15.2	0.400
Patients without diabetes (*n* = 38; 95%)	12.8 ± 4.0		28.1 ± 11.2	
Current smokers (n = 3; 7.5%)	11.5 ± 5.5	0.518	30.7 ± 27.4	0.518
Non-smokers (*n* = 37; 92.5%)	13.1 ± 3.8		26.9 ± 9.4	
Hypertension (*n* = 25; 62.5%)	13.3 ± 3.9	0.581	26.9 ± 9.6	0.391
No hypertension (*n* = 15; 37.5%)	12.2 ± 4.3		31.2 ± 13.6	
Treated with angiotensin convertase enzyme inhibitors (n = 12; 30%)	14.1 ± 3.6	0.218	26.6 ± 9.0	0.938
Not treated with angiotensin convertase enzyme inhibitors (n = 28; 70%)	12.3 ± 4.1		28.4 ± 12.0	
Treated with calcium channel blockers (*n* = 18; 45%)	12.8 ± 3.6	0.925	27.1 ± 10.3	0.545
Not treated with calcium channel blockers (*n* = 22; 55%)	12.9 ± 4.4		29.7 ± 12.2	
Treated with loop diuretics (n = 12; 30%)	15.5 ± 3.8	**0.006**	25.5 ± 7.9	0.457
Not treated with loop diuretics (n = 28; 70%)	11.7 ± 3.6		29.8 ± 12.4	
Treated with mineralocorticoid receptor antagonists (*n* = 10; 25%)	13.5 ± 4.3	0.569	29.7 ± 10.5	0.432
Not treated with mineralocorticoid receptor antagonists (*n* = 30; 75%)	12.7 ± 4.0		28.1 ± 11.7	
Treated with pentoxifylline (*n* = 20; 50%)	12.8 ± 3.6	0.758	28.4 ± 11.3	0.968
Not treated with pentoxifylline (n = 20;50%)	12.9 ± 4.5		28.7 ± 11.6	

* Mann–Whitney U-test. Statistically significant *p*-values (*p* < 0.05) are formatted in bold

Standard echocardiographic and tissue Doppler measurements were successfully performed in all patients. GLS, LA atrial strain and RA strain data were successfully obtained in 38, 39 and 37 patients, respectively. Rest of the measurements have failed due to inadequate acoustic window or foreshortening.

Correlations between clinical variables and biomarker levels are reported in Table 1. Galectin-3 and sST2 values did not show correlation with each other or with NT-proBNP levels. Neither galectin-3 nor sST2 levels showed significant correlation with age. Galectin-3 levels showed positive correlation with the duration of SSc, even in age adjusted analysis. Both Forced expiratory volume in 1 s (FEV1) and Diffusing capacity of carbon monoxide (DLCO) showed significant inverse correlation with galectin-3 levels. No significant correlations were found between sST2 levels and clinical variables. Table 2 comprises the galectin-3 and sST2 levels in various subgroups of the study population. Gender-related differences were not significant. In patients requiring loop diuretic treatment significantly higher galectin-3 levels were found.

LV ejection fraction was preserved (≥ 55%) in 39 (97.5%), whereas mildly reduced (45–54%) in 1 (2.5%) patients. LV diastolic function was impaired (Grade I diastolic dysfunction) in 17 (42.5%) patients whereas echocardiographic data suggested elevated LV filling pressure in 12 (30%) patients (all in Grade II). No patients with restrictive pattern (Grade III) were found.

RVFAC < 35%, TAPSE < 16 mm and tricuspid S < 10 cm/s were found in 1 (2.5%), 1 (2.5%) and 4 (10%) patients, respectively. Tricuspid E/e’ suggested elevated RV filling pressure in 13 (32.5%) patients. Elevated pulmonary artery systolic pressure (tricuspid Vmax> 2.8 m/s) was obtained in 11 patients with a maximum of 41 mmHg. Detailed echocardiographic data of the study population as well as correlations between galectin-3 and sST2 levels and the echocardiographic variables are reported in Tables 3 and 4.

**Table 3:** Correlations of galectin-3 (ln) and ST2 (ln) with the echocardiographic parameters of the LV and LA size and mechanics in SSc patients

Parameter		Value	Correlations with ln galectin-3		Correlations with ln galectin-3, corrected for age		Correlations with ln ST2		Correlations with ln ST2, corrected for age	
			r	p	r	p	r	p	r	p
LV ejection fraction (%)		60.6 ± 4.6	−0.273	0.089	−0.267	0.100	0.082	0.614	0.082	0.618
LV GLS (%)		−17.5 ± 2.3	**0.454**	**0.005**	**0.460**	**0.005**	0.003	0.988	− 0.007	0.969
LV mass index (g/m^2^)		93.5 ± 19.7	0.009	0.958	−0.039	0.814	−0.147	0.367	−0.184	0.262
Grade of mitral regurgitation	Mild n (%)	37 (92.5%)	**0.321**	**0.046**	**0.323**	**0.048**	−0.078	0.637	−0.080	0.632
	Moderate n (%)	3 (7.5%)								
	Severe n (%)	0 (0%)								
Mitral E (cm/s)		73.9 ± 15.7	0.006	0.970	0.028	0.867	−0.022	0.892	−0.027	0.869
Mitral A (cm/s)		72.8 ± 16.9	**0.320**	**0.044**	**0.334**	**0.038**	0.132	0.417	0.156	0.342
Septal S (cm/s)		7.3 ± 1.2	−0.224	0.166	− 0.220	0.178	0.080	0.624	0.084	0.612
Septal e’ (cm/s)		7.4 ± 2.0	−0.306	0.055	**−0.369**	**0.021**	−0.088	0.591	−0.131	0.426
Septal a’ (cm/s)		8.7 ± 1.6	−0.012	0.939	−0.018	0.912	0.181	0.263	0.183	0.265
Septal E/e’		10.5 ± 2.8	**0.375**	**0.017**	**0.380**	**0.017**	0.002	0.989	0.006	0.970
Lateral S (cm/s)		9.2 ± 1.8	−0.008	0.963	0.004	0.980	0.212	0.189	0.214	0.190
Lateral e’ (cm/s)		9.2 ± 2.8	−0.132	0.418	−0.119	0.471	0.019	0.906	0.016	0.922
Lateral a’ (cm/s)		10.6 ± 2.5	0.031	0.848	0.012	0.944	0.276	0.085	0.298	0.066
Lateral E/e’		8.7 ± 3.0	0.109	0.502	0.106	0.522	−0.063	0.699	− 0.063	0.705
Averaged S (cm/s)		8.3 ± 1.3	−0.107	0.510	−0.093	0.573	0.182	0.261	0.189	0.248
Averaged e’ (cm/s)		8.3 ± 2.2	−0.224	0.165	−0.248	0.128	−0.027	0.867	−0.045	0.784
Averaged a’ (cm/s)		9.7 ± 1.7	0.017	0.918	−0.001	0.998	0.282	0.078	0.298	0.066
Averaged E/e’		9.3 ± 2.3	0.252	0.117	0.246	0.132	−0.032	0.846	−0.030	0.856
Grade of LV diastolic function	Normal n (%)	11 (27.5%)	**0.325**	**0.040**	**0.394**	**0.013**	−0.073	0.655	−0.091	0.582
	Impaired relaxation (Grade I) n (%)	17 (42.5%)								
	Pseudonormal (Grade II) n (%)	12 (30%)								
LA Vmax index (ml/m^2^)		22.8 ± 7.4	0.016	0.925	0.001	0.994	−0.294	0.081	−0.392	0.076
LA Vmin index (ml/m^2^)		10.3 ± 5.0	0.056	0.747	0.044	0.803	−0.305	0.071	−0.410	0.078
LA Vp index (ml/m^2^)		14.2 ± 6.1	0.095	0.580	0.088	0.617	−0.237	0.164	−0.360	0.111
LA reservoir strain (%)		43.0 ± 8.7	−0.148	0.383	−0.154	0.370	−0.045	0.790	−0.005	0.978
LA conduit strain (%)		23.5 ± 6.9	−0.117	0.491	−0.137	0.426	−0.066	0.700	0.005	0.979
LA contractile strain (%)		19.5 ± 4.8	−0.102	0.549	−0.106	0.538	0.012	0.945	−0.014	0.936

Statistically significant *p*-values (p < 0.05) are formatted in bold

**Table 4:** Correlations of galectin-3 (ln) and ST2 (ln) with the echocardiographic parameters of the RV and RA size and mechanics in SSc patients

Parameter		Value	Correlations with ln galectin-3		Correlations with ln galectin-3, corrected for age		Correlations with ln ST2		Correlations with ln ST2, corrected for age	
			r	p	r	p	r	p	r	p
Pulmonary artery systolic pressure (mm Hg)		28.6 ± 7.5	0.225	0.251	0.260	0.191	−0.174	0.375	−0.188	0.348
RV basal diameter index (mm/m^2^)		18.3 ± 2.4	−0.254	0.119	−0.253	0.126	−0.015	0.928	−0.015	0.928
RV mid-cavity diameter index (mm/m^2^)		13.0 ± 2.1	−0.169	0.311	−0.169	0.318	−0.037	0.827	−0.037	0.828
RV longitudinal diameter index (mm/m^2^)		30.0 ± 3.6	−0.011	0.948	−0.011	0.948	−0.342	0.135	−0.341	0.148
Inferior vena cava (mm)		13.2 ± 3.9	−0.053	0.765	−0.052	0.775	−0.255	0.146	−0.254	0.153
Collapsibility index (%)		55.2 ± 10.7	0.134	0.449	0.134	0.457	−0.131	0.462	−0.132	0.465
RV wall thickness (mm)		5.1 ± 1.2	−0.091	0.597	−0.106	0.545	−0.051	0.766	−0.086	0.622
RV fractional area change (%)		47.9 ± 6.8	−0.138	0.423	−0.142	0.415	−0.001	0.997	0.013	0.943
Tricuspid annular plane systolic excursion (mm)		21.4 ± 2.6	−0.249	0.121	−0.243	0.136	0.315	0.248	0.321	0.284
Grade of tricuspid regurgitation	Mild n (%)	38 (95%)	0.168	0.307	0.165	0.321	−0.105	0.523	− 0.105	0.530
	Moderate n (%)	2 (5%)								
	Severe n (%)	0 (0%)								
Tricuspid E cm/s		47.4 ± 8.0	−0.073	0.658	−0.087	0.602	−0.060	0.751	−0.050	0.767
Tricuspid A cm/s		40.1 ± 8.5	**0.371**	**0.020**	**0.372**	**0.022**	−0.160	0.330	−0.162	0.331
Tricuspid S (cm/s)		12.4 ± 2.0	−0.170	0.293	−0.169	0.305	0.363	0.221	0.363	0.227
Tricuspid e’ (cm/s)		10.0 ± 2.8	−0.038	0.817	−0.009	0.956	0.006	0.972	0.001	0.996
Tricuspid a’ (cm/s)		13.1 ± 2.9	−0.100	0.539	−0.147	0.372	0.154	0.342	0.182	0.267
Tricuspid E/e’ ratio		5.2 ± 1.6	0.003	0.985	0.004	0.983	−0.001	0.995	−0.008	0.963
RA Vmax index (mL/m^2^)		19.9 ± 5.8	−0.013	0.947	0.017	0.929	−0.048	0.800	−0.101	0.602
RA Vmin index (mL/m^2^)		8.1 ± 4.0	0.227	0.227	0.245	0.201	−0.136	0.473	−0.163	0.397
RA Vp index (mL/m^2^)		14.3 ± 5.5	0.058	0.760	0.083	0.668	−0.112	0.555	−0.156	0.419
RA reservoir strain (%)		45.3 ± 9.0	−0.239	0.167	−0.236	0.179	0.218	0.209	0.229	0.193
RA conduit strain (%)		24.2 ± 6.4	−0.213	0.220	−0.210	0.233	0.199	0.253	0.220	0.212
RA contractile strain (%)		22.0 ± 6.1	−0.220	0.204	−0.231	0.188	0.065	0.713	0.056	0.753

Statistically significant p-values are formatted in bold (p < 0.05)

**Fig. 1:**
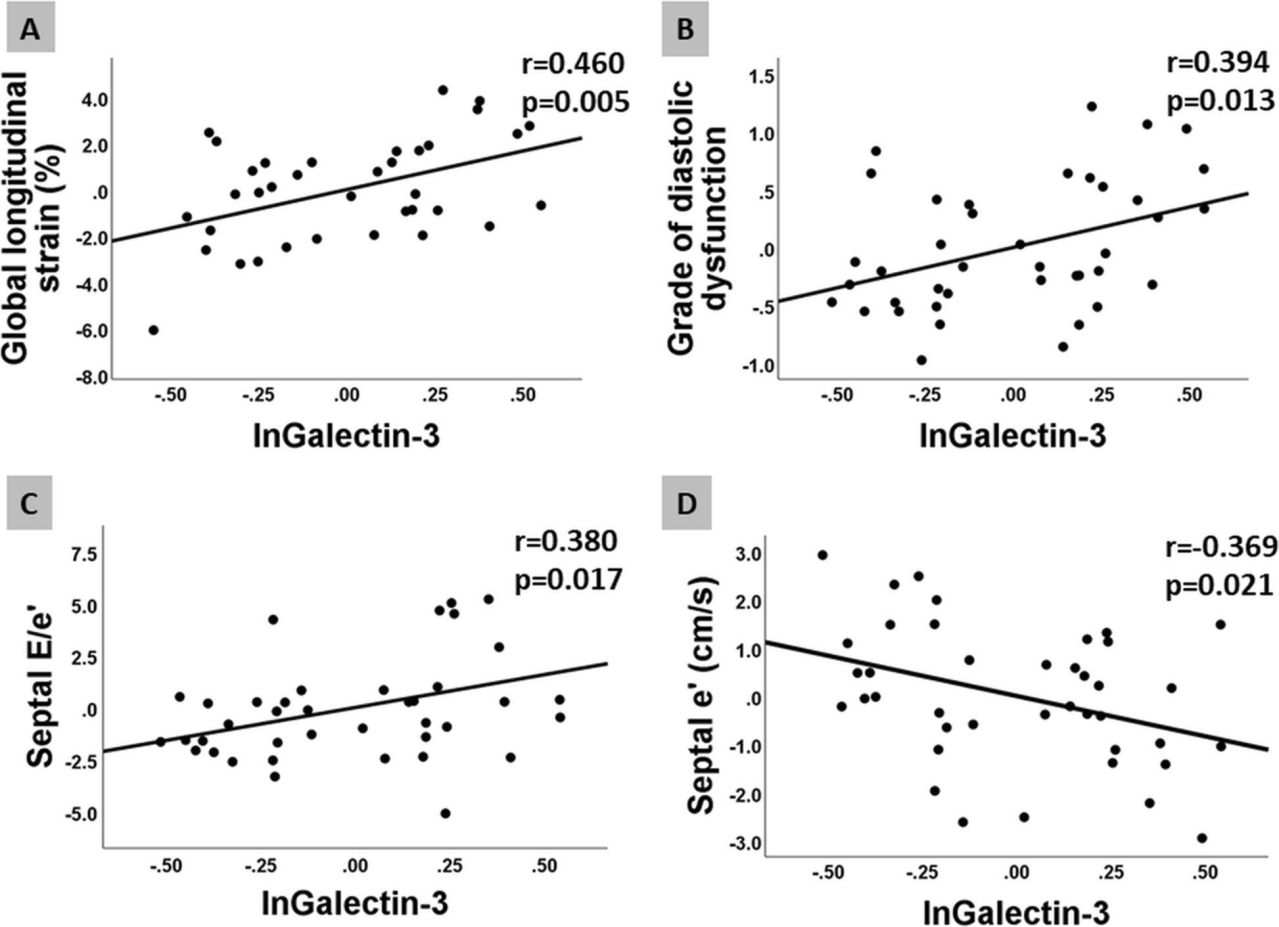
Partial regression plots demonstrate that in age adjusted analyses galectin-3 (ln) correlates with LV GLS **(A)**; with the grade of LV diastolic dysfunction **(B); **with septal E/e’**(C)** and with septal e’ **(D)**

Galectin-3 levels showed significant correlation with GLS (Fig. 1A), with the grade of LV diastolic dysfunction (Fig. 1B), with septal E/e’(Fig. 1C), and with the grade of mitral regurgitation, even in age adjusted analysis. Correlation between galectin-3 level and septal e’ became significant after age correction (Fig. 1D). No significant correlation was found between sST2 values and the echocardiographic variables.

## Discussion

Progressive cardiac fibrosis has been reported as a central aspect of the myocardial involvement in SSc [3]. Thus - although the causative role of galectin-3 and sST2 is not proved in the pathogenesis of SSc - we hypothesized that circulating biomarkers of the cardiac fibrosis may be useful in screening and early diagnosis of the cardiac manifestation in this disease. Therefore, in the present study we aimed to investigate the correlations between the levels of sST2, galectin-3 and the echocardiographic markers of the myocardial mechanics in SSc patients.

ST2 is part of the interleukin (IL)-1 receptor family which may be found on cardiac myocytes and fibroblasts. ST2 exists in two forms. The transmembrane isoform (ST2L) is responsible for the cardioprotective effect of IL-33: IL-33 has antihypertrophic and antifibrotic effects transduced by ST2L. The soluble isoform (sST2), in contrast, eliminates the cardioprotective pathway of the IL-33/ST2L interaction, by binding circulating IL-33 molecules. All clinical conditions that increase wall stress, inflammation and macrophage activation increase sST2 level and may therefore lead to an increase of cardiac fibrosis [42]. sST2 has been reported to be increased in patients with heart failure, myocardial infarction, hypertension, severe obesity, diabetes and pulmonary arterial hypertension [33, 34, 43]. In the PRIDE (ProBNP Investigation of Dyspnea in the Emergency Department) study, sST2 concentrations were higher in patients with acute heart failure and were strongly predictive of mortality at 1 year even when used together with NT-proBNP [44]. sST2 is equally prognostic in heart failure patients with preserved ejection fraction (HFpEF) as it is in those with reduced ejection fraction (HFrEF), although its concentrations are lower in patients with HFpEF [45]. Among dyspnoeic patients with and without acute heart failure, sST2 concentrations were linked to higher LV dimensions and volumes, poorer LV ejection fraction, worse right ventricular function, and higher pulmonary pressures. sST2 levels also showed correlation with the tissue Doppler-derived mitral annular e’ velocity, but not with other indices of LV diastolic function [31]. In other studies, sST2 levels did not correlate significantly with the echocardiographic markers of LV size or function but showed positive correlation with LA volume index [29], or inverse correlation with LA reservoir strain [28].

The behaviour of the IL-33/ST2 pathway has already been widely investigated is SSc. Serum IL-33 levels were elevated in SSc patients compared with healthy individuals, especially in patients with diffuse cutaneous form. IL-33 levels correlated with the extent of skin sclerosis and the severity of pulmonary fibrosis [46]. Correlations between sST2 levels and the myocardial involvement of the disease, however, have not been investigated up to now. In our present study, unlike to the previous findings, we failed to demonstrate any relationship between sST2 levels and the clinical characteristics of the disease or the echocardiographic markers of the myocardial mechanics in SSc patients.

Recent studies suggest that galectin-3 plays key role in the fibrogenesis in different organ systems, such as liver, kidney and lung [20]. In addition, galectin-3 is considered an active contributor to the development of heart failure as mediator of the myocardial fibrosis. Clinically, serum galectin-3 levels are significantly increased in patients with heart failure and are often associated with a greater risk of adverse cardiovascular events [22, 23]. Galectin-3 levels are more markedly associated with outcomes in HFpEF population compared with HFrEF patients [47].

Although the pathogenic role of galectin-3 is not proved in SSc, the potential associations between serum galectin-3 levels and the clinical features of the patients have already been investigated. Taniguchi et al. found that serum galectin-3 levels were significantly lower in the early diffuse cutaneous form of the disease compared with control subjects and showed significant correlation with total skin score. On the other hand, galectin-3 levels showed increase with the course of the disease and were higher in SSc patients with elevated right ventricular systolic pressure than in those without pulmonary hypertension [48]. Recently, Mora et al. reported lower galectin-3 expression in the skin lesions of SSc patients compared with healthy subjects. Nevertheless, relatively higher galectin-3 values were found in SSc patients with pulmonary hypertension or higher modified Rodnan skin score [49]. In addition, galectin-3 has recently been reported as an independent predictor of all-cause and cardiovascular mortality in SSc. Higher levels of galectin-3 were associated with more severe pulmonary involvement and raised inflammatory markers [50]. Our current findings also suggest that galectin-3 is a biomarker of pulmonary fibrosis in SSc. These results are in line with the work of Ho et al. They reported that in the general population elevated galectin-3 concentrations are associated with interstitial lung abnormalities including decreased lung volumes and altered gas exchange [51]. Correlations between galectin-3 levels and the echocardiographic markers of the myocardial mechanics, however, have not been investigated yet in SSc.

LV diastolic dysfunction is frequent in SSc [4–6], as it represents the primary myocardial involvement of the disease [3]. In our present study, galectin-3 levels showed significant correlation not only with the grade of LV diastolic dysfunction, but with two well defined parameters of the LV diastolic function and filling pressure: septal e’ and E/e’. Similar finding was reported by Shah at al., where higher levels of galectin-3 showed clear associations with the Doppler indices of impaired myocardial relaxation and higher filling pressure in patients with heart failure [27]. Likewise, Beltrami et al. reported significant correlation between E/e’ and galectin-3 levels in HFpEF patients [26].

Reduced LV ejection fraction is not common in SSc [7], but myocardial fibrosis may contribute to the subclinical impairment of the LV systolic function. By speckle tracking-derived 2D strain method, reduced GLS values were found in SSc patients compared to healthy subjects [8, 9]. In the study of Cameli et al., GLS showed good correlation with the extent of myocardial fibrosis in LV tissue samples obtained from heart transplantation recipients [52]. In addition, in hypertrophic cardiomyopathy patients, strong correlation was found between LV GLS and the extent of late gadolinium enhancement obtained by contrast-enhanced cardiac MRI [53]. These results suggest an unequivocal correlation between LV GLS and the severity of LV myocardial fibrosis and may explain the significant correlation found between GLS and galectin-3 levels in our study. Similarly to our findings, in the study of Hromádka et al., galectin-3 showed significant correlation with the cardiac MRI-derived parameters of the myocardial fibrosis (extracellular volume, native T1 values) and also with the speckle tracking-derived LV GLS in SSc patients [54].

Speckle tracking technique and, to a lesser extent, tissue Doppler imaging are useful in recognizing early myocardial involvement not only in the LV, but in the other cardiac chambers. Subclinical RV dysfunction was proved in SSc patients using tissue Doppler or speckle tracking measurements [10–12]. Impaired LA and RA function have also been reported in SSc, by the help of speckle tracking technique [10, 13–15]. LA reservoir strain showed significant correlation with the amount of LA wall fibrosis as assessed by cardiac MRI in patients with atrial fibrillation [55], and with the extent of LA interstitial fibrosis in patients with mitral valve disease in histopathologic specimens [56]. RV free wall strain has been reported to correlate with the extent of RV myocardial fibrosis in heart transplantation recipients [57]. Nevertheless, no correlations were found between galectin-3 levels and the tissue Doppler and strain parameters of the RV or LA and RA function in our SSc patients. These data may suggest an uncoupling between galectin-3, myocardial fibrosis, and myocardial function in these chambers, but this phenomenon requires further investigation.

### Limitations of the study

Some limitations of our study need to be acknowledged. First, in the lack of healthy control group, we could not define the cut-of value between normal and elevated serum galectin-3 or sST2 levels. Recent data suggest, however, that in the general population normal plasma concentration of galectin-3 is < 11.0 ng/ml [58], whereas the mean normal values of sST2 for males and for females are 24.9 ng/ml and 16.9 ng/ml, respectively [59].

Circulating levels of the tested fibrosis markers may not reflect the histologically proven cardiac fibrosis. Thus, circulating biomarker levels require careful interpretation in relation to myocardial involvement [60].

RV strain may better reflect the subclinical impairment of the RV systolic function than our traditional and tissue Doppler parameters. Nevertheless, in the lack of appropriate analytical software, RV strain analysis was not performed in our study.

## Conclusion

In SSc patients, galectin-3 levels show significant correlation with the parameters of LV diastolic function and with GLS, a parameter reflecting the subclinical impairment of LV systolic function. Our results suggest that galectin-3 may be a useful and simple biomarker for the screening and early identification of SSc patients with cardiac involvement. Our data does not support the use of sST2 for the same purpose.

## Data Availability

The datasets used and/or analysed during the current study are available from the corresponding author on reasonable request.

## Abbreviations

GLS: Global longitudinal strain
HFpEF: Heart failure with preserved ejection fraction
HFrEF: Heart failure with reduced ejection fraction
LA: Left atrial
LV: Left ventricular
RA: Right atrial
RV: Right ventricular
SSc: Systemic sclerosis
sST2: Soluble suppression of tumorigenicity-2
